# Agile nudge implementation to improve minority recruitment in community-based research

**DOI:** 10.3389/frhs.2026.1809432

**Published:** 2026-06-10

**Authors:** Olanrewaju Onigbogi, Malaz Boustani, Ali Ajrouch, Kebba Kah, Kolawole Okuyemi, Diana Summanwar

**Affiliations:** 1 Department of Family Medicine, Indiana University, Indianapolis, IN, United States; 2 Center for Health Innovation and Implementation Science, Indiana University, Indianapolis, IN, United States

**Keywords:** agile science, behavioral economics, community-based participatory research, implementation science, research recruitment, underrepresented populations

## Abstract

**Background:**

Recruiting underrepresented populations into community-based research remains a persistent challenge in the United States, driven in part by inequities, historical mistrust, and institutional barriers that limit access to and engagement in research. Community-Based Participatory Research (CBPR) emphasizes community engagement but offers limited guidance on how to iteratively test and refine recruitment strategies. Agile Science and specifically Agile Nudge Implementation (ANI) may provide a structured, stepwise approach to iterative improvement. The goal of the study was to determine the association between ANI strategies applied within an ongoing CBPR-informed health study and recruitment of immigrants.

**Methods:**

We conducted a quality improvement initiative embedded within the Indy Muslim Immigrants Study, a community-based survey assessing the health of Muslim immigrants from Africa and the Middle East residing in the Indianapolis metropolitan area. The study was conducted between January and August 2025. Recruitment occurred over 32 weeks and was divided into three phases: (1) a 12-week pre-implementation phase using standard CBPR-informed strategies;(2) a 12-week ANI phase; and (3) an 8-week post implementation observation phase. The ANI process followed eight-step iterative framework to design, test, and refine behavioral nudges targeting recruitment. The primary outcome was the weekly number of completed surveys. We used run chart methods to assess recruitment trends and to detect non-random variation and intervention-related changes over time.

**Results:**

A total of 709 participants completed the survey over the 32-week recruitment period. Mean weekly recruitment increased from 20.9 participants during the pre-implementation phase to 30.0 during the ANI phase (29% increase from Week 12 to 13), followed by a decline to 25.9 during the post-implementation phase, remaining above baseline. Run chart analysis demonstrated non-random variation, including a sustained shift from consecutive weeks below the baseline median prior to implementation to consecutive weeks above the median during implementation phase, consistent with meaningful improvement in recruitment performance.

**Conclusions:**

Application of the ANI process was associated with increased recruitment of Muslim immigrants, with partial sustainment recruitment after the conclusion of the formal implementation phase. Use of a structured implementation approach grounded on behavioral economics may support engagement and recruitment of immigrant and other underrepresented populations in community-based research.

## 1 Introduction

Racial and ethnic minorities remain underrepresented in community-based studies and clinical trials in the United States, despite more than three decades of policy and programmatic efforts to address this gap (e.g., the NIH Revitalization Act of 1993). This persistent underrepresentation reflects structural inequities, historical mistrust rooted in past research practices, and institutional barriers that limit access to and engagement in research. As a result, enrollment rates among immigrants and other minority populations continue to lag those of non-minority groups (1–3). For many research teams, the lived reality is familiar: protocols are approved, community partners are engaged, and yet recruitment targets are repeatedly missed (4–6).

Muslim immigrants from Africa and the Middle East exemplify this challenge. They constitute a growing but historically under-researched population with poorly documented health needs (5). Efforts to recruit this population into community-based studies have often yielded modest participant numbers, even when investigators use recommended community engagement strategies (6). Some studies suggest that the fear of being misunderstood by health care providers sometimes limits interactions with the health care system and prevents them from accessing preventative health services (7, 8). Challenges such as language differences, limited awareness of research and research participation, low trust in institutions, socioeconomic constraints, and fear of stigma have also been implicated (9, 10).

Community-Based Participatory Research (CBPR) has emerged as a leading approach to address these challenges by involving community members in all phases of the research process, improving the relevance and acceptability of research in many settings. However, despite these strengths, CBPR alone has not produced consistently large-scale improvements in minority recruitment. One reason is that while CBPR emphasizes relationships and shared governance, it offers limited guidance on how to systematically redesign recruitment processes, measure performance, and iteratively improve recruitment strategies (11, 12). Agile Science provides one such operational framework. It integrates principles from implementation science, behavioral economics, complex adaptive systems, and network science to create and sustain change in real-world settings (13). Prior studies applying agile approaches have been shown to improve processes and outcomes across inpatient and outpatient clinical settings. Within this framework, “nudges” are intentional modifications to the physical, digital, or social environment designed to influence behavior while preserving freedom of choice (13–15). These nudges draw on choice architecture and frameworks –such as MINDSPACE, and EAST. MINDSPACE s is a behavioral science framework that combines nine key drivers out of which the acronym is derived. The acronym includes the following words: messenger, incentives, norms, defaults, salience, priming, affect, commitments, and ego to influence decision-making. The EAST framework, derived from the acronym to represent easy, attractive, social, and timely, is an evidence-based approach to encourage behavior change by streamlining behavioral science to design effective, actionable nudges that simplify choices and influence decision-making (16, 17). In this study, these frameworks were applied as flexible guides and locally adapted in collaboration with community partners and Community Research Assistants (CRA), to ensure cultural relevance and acceptability within the local Muslim immigrant community.

Building on these principles, the ANI process provides a structured, eight-step method for designing, testing, and scaling evidence-informed nudges in healthcare systems (Supplementary Figure S1). Because health systems are complex, open, and adaptive networks, effective improvement requires rapid, iterative, and flexible approaches to change (16).ANI complements CBPR by providing a structured, iterative approach to testing and refining recruitment strategies. Implementation of behavioral nudges is low-cost and highly scalable compared to traditional interventions, with one study reporting as much as 100 times return in benefits for $1 spent on government-led initiatives (18). Another study reported a monetary cost as low as $23 for each at-risk patient admitted into a large hospital-based study (19). However, significant, often overlooked, human time and other costs exist, particularly regarding expert facilitation, development time, and training. These costs and the possible benefits must be considered in choosing to implement ANI interventions (20, 21).

In this study, we applied the ANI process to address a recruiting challenge in a community-based research setting. Despite using a CBPR-informed recruitment strategy, a health survey among Muslim immigrants from Africa and the Middle East in the Indianapolis metropolitan area initially did not achieve its intended weekly enrollment targets. This created a practical problem for the research team and our community partners: how to move from engagement and good intentions to a reproducible, data-driven process capable of reliably increasing recruitment. This quality improvement initiative examined the effects of integrating behavioral nudges through ANI in weekly participant recruitment real-world community-based research study. Specifically, we examined recruitment patterns before, during, and after ANI implementation using run chart methodology to detect non-random, intervention-related changes over time.

## 2 Materials and methods

### 2.1 Overview

The study was conducted as a quality improvement initiative embedded within a community health survey. The aim was to assess whether applying the ANI process to an existing CBPR-informed recruitment strategy could improve participant enrollment.

Recruitment occurred over 32 consecutive weeks and was organized into three phases: (1) a 12-week pre-implementation phase (Weeks 1–12) using standard CBPR-informed recruitment practices; (2) a 12-week ANI implementation phase (Weeks 13–24) during which the ANI process was layered onto the existing CBPR framework; and (3) an 8-week residual phase (Weeks 25–32) following the formal discontinuation of active nudges. The primary outcome was weekly recruitment, defined as the number of completed surveys per calendar week. Recruitment was monitored using run chart methodology to detect non-random changes associated with ANI.

The protocol for the Indy Muslim Immigrants Study was approved by the Indiana University Institutional Review Board (IRB 23973l). All participants provided informed consent digitally in REDCap prior to survey completion.

### 2.2 Setting

The *Indy Muslim Immigrants Study* was a CBPR-informed survey to determine the health status, access to care, and utilization of preventive care services of Muslim immigrants from Africa and the Middle East residing in the Indianapolis metropolitan area. We had support from the Indiana Muslim Community Association, a local organization with the goal of addressing social needs, promoting well-being, and strengthening community bonds, and assisting community members. We also collaborated with the leaders of ten local mosques, cultural centers, and other community organizations serving an urban region with a growing Muslim immigrant population. Recruitment took place at ten community sites which host routine events and two other venues which hosted special social events with large gatherings of Muslim immigrants.

In line with the principles of CBPR, we engaged with leaders who were identified by our partners in the Indianapolis Muslim Community Association (IMCA) and comprised of *Imams* and *Alfas* drawn from Muslim groups in Indianapolis. We had an in-person meeting two months prior and an online meeting a month prior to the commencement of the study. The purpose of these meetings was to identify the community's needs, strengths, and resources which guided the framing of the core research question, selection of study sites, identification of variables, and refining of study methods. In addition, we had two follow-up meetings in each mosque or community group which hosted the study. These meetings were designed to obtain feedback and ensure success during the study. In addition, we took into consideration the importance of trust, privacy, modesty, and gender concordance in setting up our research environment for data collection. In view of this, we recruited only CRA workers who were practicing Muslims to ensure that all cultural considerations around appropriate language, gestures and accommodation of prayer times were considered ab-initio and incorporated into the research protocol. The study team comprised ten CRAs, all of whom were members of the local Muslim immigrant community. CRAs were selected based on linguistic proficiency, community standing, and interest in research participation. Prior to participant recruitment, all CRAs completed structured training on human participants’ protection, informed consent procedures, use of REDCap, and standardized survey administration. In addition, we took into consideration the importance of trust, privacy, modesty, and gender concordance in setting up our research environment for data collection. In view of this, we recruited only CRA workers who were practicing Muslims to ensure that all cultural considerations around appropriate language, gestures and accommodation of prayer times were considered ab-initio and incorporated into the research protocol.

The survey instrument was a self-administered questionnaire delivered electronically via REDCap and available in English, Somali, and Arabic. Participants completed the survey on study-provided tablets or their own smartphones after providing consent. The survey was adapted from the Population Assessment of Tobacco and Health (PATH) Wave 7 Questionnaire and included four domains: general health, health-related risk factors, healthcare access and utilization, and socio-demographics and religiosity.

### 2.3 ANI process

After 12 weeks of recruitment using standard CBPR-informed strategies that did not meet the predefined target of 24 completed surveys per week, the research team introduced the ANI process at Week 13. ANI process is an eight-step, Agile Science-based method for designing, testing, and scaling behavioral nudges in real-world settings (Table 1). The process was implemented across two overarching phases: a planning phase (Steps 1–4) and an execution phase (Steps 5–8). The goal of ANI was to identify specific behavioral bottlenecks in recruitment, design nudges to address them, test nudges in short implementation sprints, and then codify effective strategies into minimally standard operating procedures (mSOP). Sprints are part of ‘learning cycles’ which are short, fixed-length time-boxed iterations designed to build knowledge, gain feedback, and reduce risk. They empower teams to validate assumptions via prototypes or incremental value delivery, adapting to changes quickly rather than following rigid plans (16, 17).

**Table 1:** Behavioral mechanisms and intended effects of nudges.

Nudge	Behavioral Mechanism	MINDSPACE Element(s)	EAST Principle(s)	Intended Effect
Retention of CRAs from the same cultural background as participants (Week 13–24)	Trusted messengers for the study.	M–Messenger (credible community context)	A-Attractive	Makes study easily acceptable among participants
Flyers in mosque changing rooms and community centers (Week 13–16)	Increased visibility and contextual salience of study in familiar, trusted spaces	S – Salience M – Messenger (credible community context)	E – Easy A – Attractive	Makes study participation novel and relevant in daily community routines
Weekly performance graphs emailed to CRAs (Week 13–16)	Feedback and social comparison	I – Incentives E – Ego S – Salience	T – Timely A – Attractive	Encourages consistent recruitment effort through peer accountability
$25 incentive for top recruiter (Week 13–16)	Monetary reward reinforces competitive motivation	I – Incentives E – Ego	A – Attractive S – Social	Promotes performance through loss aversion
Recognition certificate for ≥3 recruits/week (Week 13–16)	Ego and recognition serve as non-monetary motivation	E – Ego I – Incentives (non-financial)	A – Attractive S – Social	Reinforces expectation and pride in performance
Withdrawal of incentives and introduction of loss-aversion framing (Week 17–20)	Loss aversion: fear of losing recognition drives persistence	I – Incentives E – Ego	A – Attractive S – Social	Sustains motivation to avoid removal of rewards
Distinctive CRA dress codes (Week 17–20)	Environmental modification signaling identity and accountability	P – Priming E – Ego S – Salience	A – Attractive S – Social	Increases visibility and promotes trust
Large QR code posters in mosque ablution rooms (Week 21–24)	Artefactual reminders-continuous cue for recall and ease of participation	S – Salience	E – Easy T – Timely	Encourages engagement by reducing effort
Weekly encouragement messages to CRAs and community members (Week 21–24)	Social norms and emotional reinforcement	A – Affect N – Norm E – Ego	S – Social T – Timely	Creates collective behavior

#### 2.3.1 Planning phase (steps 1–4)

##### 2.3.1.1 Step 1: confirming demand for a nudge and identifying opportunities

During the first 12 weeks of recruitment, weekly enrollment consistently fell short of the predefined target of 24 complete surveys per week, despite standard CBPR engagement. This persistent gap was interpreted as evidence of “demand for a behavioral nudge”, an observation which has been identified in previous studies (22, 23).In response, the research team and community partners, particularly *Imams* and *Alfas*, reviewed cultural factors influencing participation and identified key opportunities to improve awareness and visibility of CRA activities at community sites by conducting one-on-one outreaches to potential participants, in-person connections at community hubs, and the creation of materials to which participants can easily relate. Guided by the “messenger” principle of the MINDSPACE framework, the research team reaffirmed and leveraged the fact that CRAs were trusted members of the same religious and cultural communities to attract the attention of potential participants.

##### 2.3.1.2 Step 2: studying behavior and selecting evidence-based nudges

We conducted a focused assessment of recruitment behavior from the perspective of both potential participants and CRAs. These insights were used to identify barriers and facilitators to participation and select behaviorally informed nudges described in the literature (2). In Week 12, selected nudges were adapted to the local context in partnership with community leaders, with focus on the design of guidance tools and communication methods.

##### 2.3.1.3 Step 3: developing evaluation and termination plans

Before initiating the ANI phase, the team specified performance metrics and *a priori* decision rules to guide evaluation and termination. Weekly recruitment, defined as the number of surveys completed per calendar week, was selected as primary metric, with a target of a 20% cumulative increase during Weeks 13–24 compared with the pre-implementation phase (24). Termination criteria included (1) lack of improvement over four consecutive weeks or (2) evidence of burden or unintended negative consequences for CRAs or participants. Evaluation plans were aligned with run chart methods to distinguish random variation from meaningful change.

##### 2.3.1.4 Step 4: localizing the nudge and mapping the process

The existing recruitment process was mapped in detail, including CRA scheduling, site assignment, invitation scripts, and follow-up practices. This process map was used to identify leverage points at which nudges could be introduced. Nudge selection was informed by established behavioral frameworks such as MINDSPACE and EAST (13–17).

#### 2.3.2 Execution phase (steps 5–8)

##### 2.3.2.1 Step 5: executing implementation sprints

ANI was implemented through short sprints, each introducing one or more nudges. Implemented nudges included redesigned flyers and QR codes placed in high-traffic locations; a standardized CRA dress code intended to enhance visibility and trust; weekly performance feedback and recognition for CRAs; and financial incentives framed using behavioral economics principles, such as loss aversion. Loss aversion, which is defined as the tendency to weigh losses more heavily than equivalent gains, has demonstrated effects in other contexts (25). Table 1 summarizes the behavioral mechanisms underlying each nudge and their intended effects on recruitment. CRAs from the same cultural background as participants were retained from Week 13–24 to ensure that the study remained acceptable among participants. Activities such as placement of flyers in changing rooms inside the mosques and creation of weekly performance graphs for CRAs took place in Week 13–16. These activities had the objectives of making the study relevant to routines activities and encouraging peer accountability. The activities from Week 13–16 also included a gift of $25 as an incentive to the top recruiter for each week while recognition certificates were issued for CRAs who recruited up to 3 participants per week with the view of promoting overall performance. Week 17–20 involved the threat of withdrawal of incentives and use of specified color matching dress codes for recruitment events that involved groups of CRA. These activities had the objectives of sustaining motivation and increasing the visibility of recruitment teams. In addition, posters with large QR codes were pasted on information boards of mosques and weekly encouragement messages were sent via short message service (SMS) were sent by the PI to the phones of CRAs and *Imams* or onward transmission to congregants. The goal of these activities in Week 21–24 was to encourage engagement and collective behavioral change. Continuous monitoring of weekly recruitment and collection of qualitative feedback from CRAs allowed the team to modify, intensify, or discontinue nudges in real time based on observed performance and frontline experience. The feedback was obtained on a weekly basis during virtual rendezvous meetings from 4–5pm every Friday throughout the course of the study. Examples of questions in the feedback sessions with CRA and community leaders include the proportion of eligible respondents who eventually agreed to participate in the survey, the number of persons who commenced but chose to drop-out of the survey and the main reasons given for attrition.

##### 2.3.2.2 Step 6: monitoring performance

Recruitment data were plotted weekly on a run chart with the median line calculated across the 32-week period. Each sprint incorporated rapid testing and modification cycles using pre-defined outcome measures for weekly and monthly recruitment goals. Regular review meetings allowed continuous interpretation of patterns, including immediate and delayed effects of nudges, and real-time adjustments to nudge design.

##### 2.3.2.3 Step 7: assessing organizational impact

The team evaluated the broader impact of ANI on the recruitment process and organizational routines, including CRA workload, perceived fairness of incentives, and relationships with community partners. Feedback for organizational impact was obtained through informal debriefs and structured reflections with CRAs and community partners.

##### 2.3.2.4 Step 8: developing minimally standard operating procedures

Nudges that demonstrated effectiveness and acceptability were consolidated into mSOPs to support future recruitment efforts. These mSOPs included specific guidance on CRA selection and training, design and placement of recruitment materials, incentive structures, and performance feedback and evaluation processes. The goal was to develop a replicable and adaptable implementation package that could be applied across diverse community settings (Supplementary Table 1).

### 2.4 Study measures and analysis

The primary outcome measure was weekly recruitment, over the 32-week study period. All completed surveys recorded in REDCap during the study period were included in the analysis, regardless of recruitment site.

The initiative used a time-series quality improvement design. Weekly recruitment data were collected prospectively across three phases (pre-implementation, ANI implementation, and residual) with introduction of the ANI process at a single, clearly defined time point (Week 13). No randomization or comparison group was used; instead, the analytic focus was on detecting non-random changes in recruitment patterns over time associated with the introduction of ANI.

**Figure 1:**
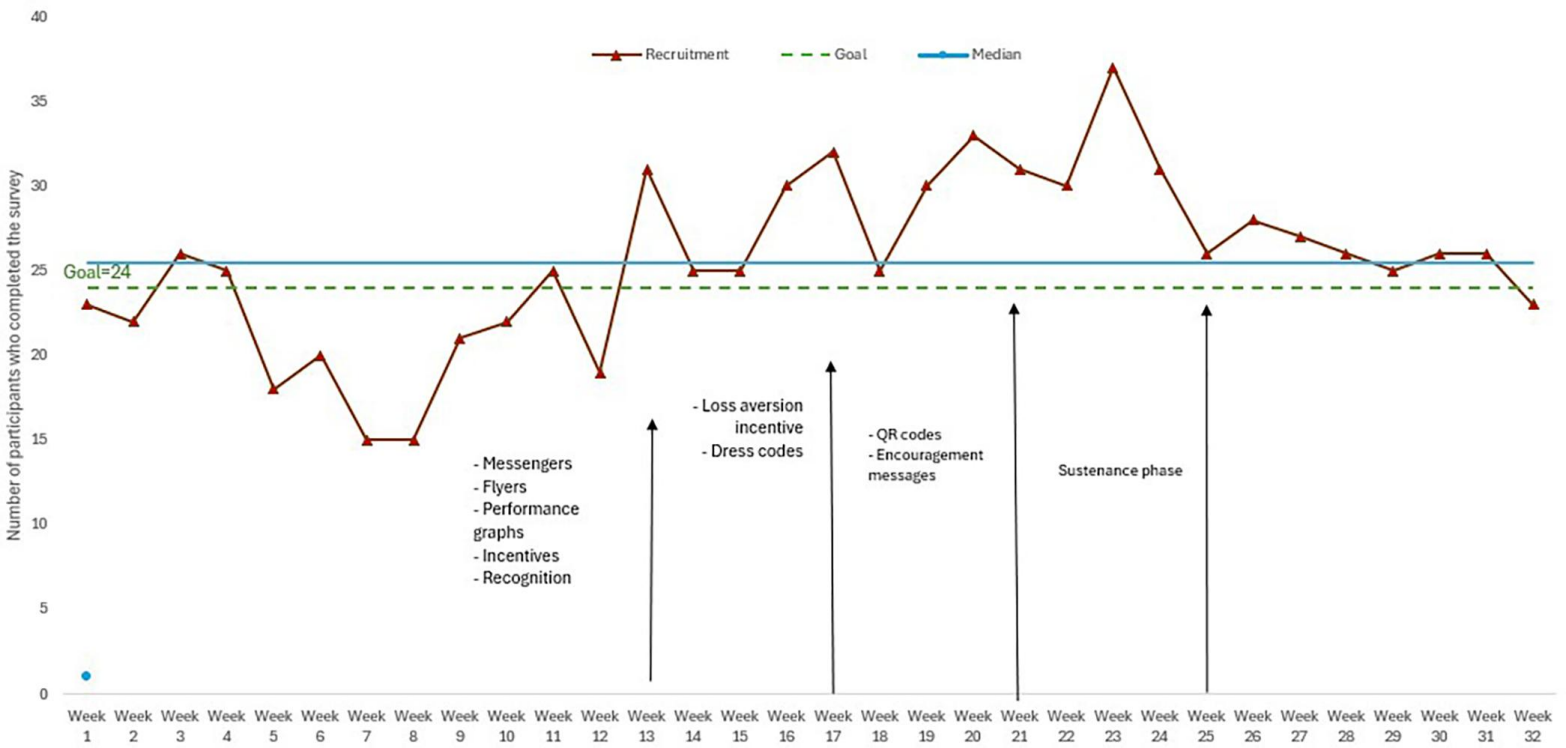
Run Chart of recruitment and interventions.

Run charts were constructed with calendar week on the *x*-axis and weekly recruitment on the *y*-axis to visualize process variation and identify non-random trends over time (Figure 1). Under conditions of random variation, data points are expected to fluctuate around the median without sustained patterns (26). Standard run chart rules were applied to identify non-random patterns, including shifts (six or more consecutive points above or all below the median), trends (five or more consecutive points consistently increasing or decreasing), and runs (too few or too many crossings of the median compared with what would be expected by chance) (27). A run chart is a line graph used to visualize data over time to identify process improvements, trends, shifts of cycles. It typically utilizes a median as a center line to separate common-cause from special-cause variation and plots data chronologically on the horizontal axis and the measured indicator on the vertical axis, helping to distinguish real improvement from random noise (27). Interpretation followed established quality improvement to determine whether observed changes were likely attributable to the ANI process rather than random variation.

## 3 Results

The study was conducted from January through August 2025. A total of 709 participants were recruited over the 32-week period. The mean (SD) age was 23.5 (5.0) years; 50.1% were male, 55.5% were married, and 50.9% with completed high school education (Table 2). Recruitment trends are described across three phases: a twelve-week pre-implementation phase (Weeks 1–12), a twelve-week ANI phase (Weeks 13–24), and an eight-week residual phase following completion of active nudges (Weeks 25–32).

**Table 2:** Demographic characteristics of participants.

Characteristic	Value (%)
Age group, *n* (%)	
Older adolescents (18–20)	123 (17.3)
Young adults (21–24)	191 (26.9)
Adults (25–59)	246 (34.8)
Older adults (60+)	149 (21.0)
Gender, *n* (%)	
Male	355 (50.1)
Female	354 (49.9)
Marital status, *n* (%)	
Single	221 (31.2)
Married/partnered	390 (55.0)
Divorced/widowed	78 (11.0)
Cohabiting	20 (2.8)
Highest educational level, *n* (%)	
No formal education	141 (19.9)
Middle school	207 (29.2)
High school/some college	265 (37.4)
University or postgraduate degree	96 (13.5)
Employment status, *n* (%)	
Unemployed	102 (14.4)
Student	161 (22.7)
Employed	242 (34.1)
Self-employed	127 (17.9)
Retired	77 (10.9)

During the pre-implementation phase, mean weekly recruitment was 20.9 participants (range 18–26), and the predefined target of 24 participants per week was not met on a sustained basis. At Week 13, the ANI process was introduced, incorporating environmental and salience cues, structured feedback to CRAs, and incentive-based nudges. Mean weekly recruitment increased to 30.0 participants during the ANI phase, with an immediate increase to 31 participants in Week 13, representing a 29% increase compared to Week 12. Subsequent implementation sprints that layered loss-aversion framing and standardized CRA dress codes were followed by several weeks in which weekly recruitment exceeded the median of 25.5 participants. During the residual phase, after formal discontinuation of nudges in Week 24, mean weekly recruitment declined slightly to 25.9 participants but remained above the pre-implementation average.

Run chart analysis of the recruitment data demonstrated non-random, intervention-associated variation in recruitment over time (Figure 1). During the pre-implementation phase, nine consecutive data points fell below the median, indicating a sustained downward shift. Following the introduction of ANI, six consecutive data points fell above the median, meeting criteria for a sustained upward shift in weekly recruitment. A sequence of five consecutive increases between Weeks 7 and 11 met criteria for a positive trend. There were neither too few nor too many runs across the median compared with what would be expected by chance, and all observed values remained within expected process limits. Collectively, these patterns indicate meaningful, non-random improvement in recruitment with the ANI process (26, 27).

## 4 Discussion

In this community-based survey of Muslim immigrants from Africa and the Middle East, application of the ANI process was associated with substantial improvement in participant recruitment. Mean weekly enrollment increased from 21 participants during the pre-implementation phase to 30 participants during the ANI phase, with partially sustained gains into the residual period. Run-chart analysis demonstrated non-random variation consistent with intervention-associated improvement. These findings suggest that minority recruitment in community-based research can be enhanced not only through engagement and partnership, as emphasized in CBPR, but also through a structured, iterative redesign of the physical, social, and organizational environments that shape participant behavior. The 29% improvement in recruitment rates seen in our study, agrees with similar ones to determine the effectiveness of utilizing a texting nudge to enhance post-agreement recruitment of participants with cognitive impairments, introduction of standard operating procedures led to doubled 6-month average recruitment rate (28) while another study to enhance enrollment of pregnant women in a smoking cessation trial showed that behavioral nudges increased enrollment by as much as 63% (29). Our study also shows that even though CBPR interventions increase enrollment rates in minorities (30, 31). adopting ANI techniques after prior use of CBPR techniques have failed to achieve the required recruitment expectations may be more promising. The observed effects of ANI can also be interpreted using the American Thoracic Society (ATS) framework for improving minority participation in research, which emphasizes interventions across four levels—individual, interpersonal, institutional/system, and federal/policy—to addressed persistent barriers to enrollment (32). In our study, the behavioral nudges designed and deployed through ANI operated across multiple levels simultaneously, creating a layered intervention that likely contributed to the observed recruitment patterns. This multilevel approach helps explain both the size of the recruitment increase and its partial sustenance, compared with prior U.S.-based interventions that typically focus on a single recruitment strategy (33, 34). By translating behavioral theory into mSOPs, this work contributes practical tools for researchers and policy makers seeking to improve minority recruitment in similar community-based settings. Although all active nudges concluded at the end of Week 24, certain environmental artifacts, such as flyers and printed QR codes displayed in community settings, remained in place. These elements may have continued to exert a residual priming effect during the post-implementation phase, potentially supporting recruitment levels that remained above the pre-implementation mean.

At the individual level, we tried to address systemic barriers such as logistical hurdles (e.g., transportation and childcare) and our resource limitations by introducing environmental and salience cues. Placement of flyers and QR codes placed in mosques and community centers may have also increased study visibility and reduced access friction, while plain-language materials and weekly reminder messages to CRAs may have contributed to maintained attention and motivation. Similar strategies have been associated with improved recruitment in other studies (35–37).

At the interpersonal level, mistrust and weak social connection were addressed through feedback, recognition, and social identity cues. Close collaboration between the investigators and CRAs, coupled with weekly performance summaries, likely fostered transparency, shared accountability, and a sense of collective purpose. Recognition certificates and standardized dress codes during outreach events may have reinforced trust by signaling professionalism and cultural alignment. Some studies have reported on the extent to which community partners value interpersonal trust as a core component of their desire for continued collaboration emphasizing the need for all partners to deliver on their promises (38, 39). Prior research also suggests that our strategies can strengthen social credibility and enhance recruitment among underrepresented populations (40, 41). It is also important to highlight the human and time costs associated with conceptualizing, planning, and implementing the ANI intervention must be considered in decision-making about its feasibility. These costs have been identified in previous studies and serve as a reminder for people interested in using the ANI approach to ensure that the expected benefits will outweigh the costs (20, 21).

At the institutional/system level, ANI functioned as an intervention on recruitment processes themselves. While some studies highlight challenges in implementing process-level changes (42, 43), the structured introduction of monetary incentives, followed by loss-aversion framing and intrinsic recognition, appeared to support consistent recruitment efforts while embedding real-time learning cycles (25, 44). The sustained sequence of data points above the median following introduction of loss-aversion message suggests that this component may have been particularly influential. Finally, although this study was conducted entirely at the community level, the ANI process aligns with the policy/federal level of the ATS framework by operationalizing equity goals into measurable targets, accountability structures, and scalable mSOPs. By establishing explicit recruitment targets, monitoring performance weekly, and codifying effective practices, ANI illustrates how behavioral design can create bottom-up mechanisms that complement top-down equity mandates (37, 38).

### 4.1 Limitations

Our study has several limitations that should be considered when interpreting the findings. First, as a quality improvement initiative conducted across multiple community sites within a single metropolitan area and part of a specific CBPR-informed project, we cannot fully account for external factors that may have influenced recruitment over time. Weather conditions, local or national events, and religious or community festivities could have affected attendance at recruitment sites and the availability of potential participants in ways that coincided with, but were not caused by, the ANI process. Future studies could strengthen causal inference by prospectively documenting such contextual factors and integrating quantitative time-series analysis with parallel qualitative process evaluation. In addition, we note the limitations due to non-randomization of community recruiters into different conditions of ANI to determine the effect size in each group or using a reverse design methodology in which reverting to the baseline intervention after the ANI would have shown if the changes were spurious. These methods can be adopted by other researchers to increase their study's reliability. Moreover, our study was not designed to disaggregate data from participants who were recruited directly from our community initiatives from others who were recruited via online initiatives such as Muslim group online portals and social media.

Furthermore, the quasi-experimental, time-series design lacked randomization or a concurrent comparison group. Although run chart analysis demonstrated non-random variation consistent with intervention-related improvement, we cannot definitively attribute all observed changes in recruitment to ANI alone. Secular trends, unmeasured changes in community dynamics, or evolving CRA experience may have contributed to the observed patterns. In addition, multiple nudges were introduced in overlapping sprints, limiting our ability to isolate the independent effects of individual components (e.g., loss-aversion incentives vs. dress code or QR code placement).

Finally, the generalizability of these findings is limited. The intervention was implemented in one U.S. metropolitan area and focused on Muslim immigrants from Africa and the Middle East. Other immigrant groups, geographic regions, or research contexts may require different combinations of nudges and adaptations of the ANI process. Moreover, our primary outcome was weekly recruitment volume; we did not formally evaluate implementation outcomes such as acceptability, feasibility, cost, or sustainability. Neither did we assess whether changes in recruitment translate into differences in long-term engagement or health outcomes. Therefore, a mixed methods approach to the study would have been helpful to gain additional insight into some of our findings.

These limitations highlight the need for additional studies evaluating the ANI process and related Agile Science approaches across diverse populations and settings, ideally using rigorous comparative designs and a broader set of implementation outcomes.

## 5 Conclusion

Applying the ANI process within a CBPR-informed community health study was associated with a meaningful increasement in participant recruitment, with gains partially sustained after active implementation ended. Weekly enrollment increased from approximately 21 participants during the pre-implementation phase to 30 during the ANI phase and remained above baseline in the residual phase, with run chart analysis indicating non-random improvement.

This study contributes evidence that a structured, ANI approach can be used to improve recruitment in community-based research. Future studies should evaluate the application of ANI in other population and study designs, and assess scalability, sustainability, and cost, as well as its alignment with broader institutional and policy efforts to reduce inequities in research participation.

## Data Availability

The raw data supporting the conclusions of this article will be made available by the authors, without undue reservation.
