# Supplementary material for: Agile nudge implementation to improve minority recruitment in community-based research
Source: Front Health Serv. 2026 Jun 10;6:1809432. doi: 10.3389/frhs.2026.1809432 (PMC13290861; doi:10.3389/frhs.2026.1809432)
Supplement: Supplementary file 1 [file Table1.docx]

## Table 1. Behavioral Mechanisms and Intended Effects of Nudges

| **Nudge** | **Behavioral Mechanism** | **MINDSPACE Element(s)** | **EAST Principle(s)** | **Intended Effect** |
| --- | --- | --- | --- | --- |
| Retention of CRAs from the same cultural background as participants (Week 13-24) | Trusted messengers for the study. | M–Messenger (credible community context) | A-Attractive | Makes study easily acceptable among participants |
| Flyers in mosque changing rooms and community centers (Week 13-16) | Increased visibility and contextual salience of the study in familiar, trusted spaces | S – Salience  M – Messenger (credible community context) | E – Easy  A – Attractive | Makes study participation novel and relevant in daily community routines |
| Weekly performance graphs emailed to CRAs  (Week 13-16) | Feedback and social comparison | I – Incentives  E – Ego  S – Salience | T – Timely  A – Attractive | Encourages consistent recruitment effort through peer accountability |
| $25 incentive for top recruiter  (Week 13-16) | Monetary reward reinforces competitive motivation | I – Incentives  E – Ego | A – Attractive  S – Social | Promotes performance through loss aversion |
| Recognition certificate for ≥3 recruits/week (Week 13-16) | Ego and recognition serve as non-monetary motivation | E – Ego  I – Incentives (non-financial) | A – Attractive  S – Social | Reinforces expectation and pride in performance |
| Withdrawal of incentives and introduction of loss-aversion framing (Week 17-20) | Loss aversion:  fear of losing recognition drives persistence | I – Incentives  E – Ego | A – Attractive  S – Social | Sustains motivation to avoid removal of rewards |
| Distinctive CRA dress codes (Week 17-20) | Environmental modification signaling identity and accountability | P – Priming  E – Ego  S – Salience | A – Attractive  S – Social | Increases visibility and promotes trust |
| Large QR code posters in mosque ablution rooms (Week 21-24) | Artefactual reminders—continuous cue for recall and ease of participation | S – Salience | E – Easy  T – Timely | Encourages engagement by reducing effort |
| Weekly encouragement messages to CRAs and community members  (Week 21-24) | Social norms and emotional reinforcement | A – Affect  N – Norm  E – Ego | S – Social  T – Timely | Creates collective behavior |
