# Supplementary material for: Agile nudge implementation to improve minority recruitment in community-based research
Source: Front Health Serv. 2026 Jun 10;6:1809432. doi: 10.3389/frhs.2026.1809432 (PMC13290861; doi:10.3389/frhs.2026.1809432)
Supplement: Supplementary file 2 [file Table2.docx]

## Table 2. Demographic characteristics of participants

| Characteristic | Value (%) |
| --- | --- |
| **Age group, n (%)** |  |
| Older adolescents (18–20) | 123 (17.3) |
| Young adults (21–24) | 191 (26.9) |
| Adults (25–59) | 246 (34.8) |
| Older adults (60+) | 149 (21.0) |
|  |  |
| **Gender, n (%)** |  |
| Male | 355 (50.1) |
| Female | 354 (49.9) |
|  |  |
| **Marital status, n (%)** |  |
| Single | 221 (31.2) |
| Married / partnered | 390 (55.0) |
| Divorced / widowed | 78 (11.0) |
| Cohabiting | 20 (2.8) |
|  |  |
| **Highest educational level, n (%)** |  |
| No formal education | 141 (19.9) |
| Middle school | 207 (29.2) |
| High school / some college | 265 (37.4) |
| University or postgraduate degree | 96 (13.5) |
|  |  |
| **Employment status, n (%)** |  |
| Unemployed | 102 (14.4) |
| Student | 161 (22.7) |
| Employed | 242 (34.1) |
| Self-employed | 127 (17.9) |
| Retired | 77 (10.9) |
