# Supplementary material for: Agile nudge implementation to improve minority recruitment in community-based research
Source: Front Health Serv. 2026 Jun 10;6:1809432. doi: 10.3389/frhs.2026.1809432 (PMC13290861; doi:10.3389/frhs.2026.1809432)
Supplement: Supplementary file 3 [file Datasheet1.docx]

# Supplemental Material 1

# Minimally Standard Operating Procedure (mSOP): Community-Based Participant Recruitment Using Agile Nudge Implementation

## Purpose

To provide a standardized, evidence-based procedure for recruiting participants from underrepresented communities using the Agile Nudge Implementation process. This mSOP ensures culturally appropriate engagement, consistent performance monitoring, and iterative improvement in recruitment outcomes.

## Scope

This mSOP applies to all study personnel involved in community-based recruitment activities, including the Principal Investigator, Co-Investigators, Community Research Leaders, Community Research Assistants, and members of the Community Advisory Board. It is suitable for recruitment in faith-based or community settings where trust, accessibility, and behavioral engagement are critical.

## Responsibilities

- Principal Investigator: Provides overall leadership, approves recruitment materials, monitors performance metrics, and ensures compliance with ethical standards.
- Community Research Leader: Serves as liaison between the study team and community leaders, ensuring cultural alignment and community trust.
- Community Research Assistants: Conduct outreach, administer surveys, and document recruitment activities following this mSOP.
- Community Advisory Board: Offers guidance on cultural sensitivity, trust-building, and community-specific adaptations.
- Data Manager/Analyst: Monitors weekly recruitment data and communicates performance metrics to the PI and CRAs.

## Procedure

### Step 1. Identify Recruitment Sites and Partnerships

Engage local community and faith-based leaders (e.g., imams, pastors, or community heads) to secure endorsement and access to recruitment venues such as mosques, community centers, or markets. Establish a Community Advisory Board to review materials, guide trust-building, and provide ongoing feedback. Hire and train Community Research Assistants who reflect the community’s demographic and cultural profile and can utilize popular community events such as food and culture festivals to maximize recruitment.

### Step 2. Prepare Recruitment Materials

Develop flyers, posters, and digital media following plain-language and cultural relevance guidelines. Ensure materials include QR codes for easy access to study information and surveys. Translate materials into the predominant community languages where applicable and obtain ethical approvals for all recruitment materials.

### Step 3. Implement Behavioral Nudges (Choice Architecture)

| **Nudge Description** | **Nudge Type / Associated Cognitive Bias** | **Implementation Guidance** |
| --- | --- | --- |
| Recruitment of CRAs from the same cultural background as participants  Flyers in mosques/community centers | Messenger, Commitments  Salience, Priming/Facilitation, Anchoring | Encourage CRAs to recruit first among their family and social networks  Place in high-traffic, familiar areas (e.g., entrances, changing rooms). |
| Weekly Community Research Assistants performance feedback | Priming, norm /Anchoring, Social comparison, accountability | Email graphical dashboards showing weekly recruitment vs. target. |
| $25 incentive for top recruiter | Incentive, Priming/ Feedback, Norms, Anchoring, Affect | Offer publicly announced rewards for top performance each week. |
| Recognition certificates | Ego, incentive/ social recognition | Provide non-monetary certificates for ≥3 weekly recruits. |
| Distinct Community Research Assistants dress codes | Priming, salience / Identity, availability | Require standardized apparel to enhance credibility. |
| Loss-aversion framing | Priming, Salience, affect/ Loss aversion | Frame recognition as something to sustain |
| QR code posters in ablution rooms | Priming, salience/Facilitation, availability | Facilitate immediate action (scan and enroll). |
| Encouragement messages | Priming, salience, affect/ Framing, social norm | Send weekly motivational messages to CRAs and community members. |

### Step 4. Run Agile Recruitment Sprints

Divide implementation into one-month sprints. Review weekly data (recruitment rate, response completeness). Adapt nudges based on performance trends –add, remove, or reframe messages as needed. Document all changes in a Sprint Log.

### Step 5. Monitor and Evaluate Recruitment Performance

Define lead measures (number of recruits per week, CRA engagement rates, survey completion rates). Define lag measures (monthly recruitment growth, retention, diversity metrics). Identify success as ≥20% monthly recruitment growth or ≥50% above baseline.

### Step 6. Quality Control and Data Management

Track completeness of REDCap questionnaires via automated monitoring. Investigate missing data weekly. Ensure participant compensation ($30 gift card) is documented and auditable.

### Step 7. Termination and Iteration Criteria

Pause and reassess if total weekly recruitment falls below 12 participants for four consecutive weeks. Discontinue ineffective nudges and document lessons learned.

### Step 8. Develop Standard Operating Package for Scale-Up

Once validated, consolidate into a Business or Implementation Package, including templates for materials, training manuals, and monitoring tools.

## Documentation

- Weekly CRA performance reports
- Community engagement logs
- Nudge implementation tracker
- Monthly PI performance summary
- Participant compensation records
