# Supplementary figures and images for: Agile nudge implementation to improve minority recruitment in community-based research
Source: Front Health Serv. 2026 Jun 10;6:1809432. doi: 10.3389/frhs.2026.1809432 (PMC13290861; doi:10.3389/frhs.2026.1809432)

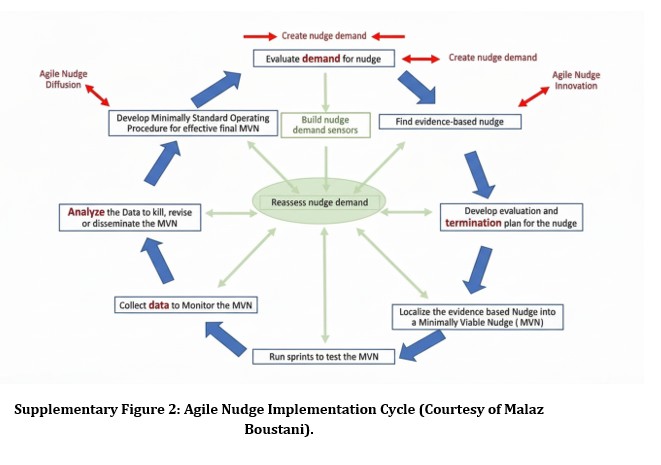

Supplement: Supplementary file 4 [file Image1.jpg]
